# Prognostic value of Onodera’s nutritional index for intermediate- and high-risk gastrointestinal stromal tumors treated with or without tyrosine kinase inhibitors

**DOI:** 10.1186/s12957-021-02345-9

**Published:** 2021-08-03

**Authors:** Feng Wang, Tingting Tao, Heng Yu, Yingying Xu, Zhi Yang, Xuefeng Xia, Meng Wang, Liang Zong, Wenxian Guan

**Affiliations:** 1 Department of General Surgery, Drum Tower Clinical Medical College of Nanjing Medical University, Nanjing, Jiangsu Province People’s Republic of China; 2 Department of General Surgery, Nanjing Drum Tower Hospital, The Affiliated Hospital of Nanjing University Medical School, Nanjing, Jiangsu Province People’s Republic of China; 3 Department of General Surgery, Yizhen People’s Hospital, Clinical Medical College, Yangzhou University, Yangzhou, Jiangsu Province People’s Republic of China; 4 Department of Gastrointestinal Surgery, Changzhi People’s Hospital, The Affiliated Hospital of Changzhi Medical College, Changzhi, Shanxi Province People’s Republic of China

**Keywords:** Gastrointestinal stromal tumor, Neutrophil-to-lymphocyte ratio, Platelet-to-lymphocyte ratio, Onodera’s prognostic nutritional index, Propensity score matching, Prognostic marker

## Abstract

**Background:**

Immunoinflammatory and nutritional markers, such as the peripheral blood neutrophil-to-lymphocyte ratio (NLR), platelet-to-lymphocyte ratio (PLR) and Onodera’s prognostic nutritional index (OPNI), have gained considerable attention and have been preliminarily revealed as prognostic markers of gastrointestinal stromal tumors (GISTs).

**Methods:**

In this study, we first investigated the prognostic value of OPNI in GISTs treated with or without TKIs based on the propensity score matching (PSM) method. All of the patients had received surgical resection for primary GIST, and data from 2010 to 2018 were initially and retrospectively identified from our gastrointestinal center. Recurrence-free survival (RFS) was calculated by the Kaplan–Meier method and compared by the log-rank test.

**Results:**

The patients were divided into groups treated and not treated with TKIs, and we used the propensity score matching method to homogenize their baseline data. Multivariate Cox proportional hazard regression models were applied to identify associations with outcome variables. A total of 563 GISTs were initially chosen, and 280 of them were included for analysis under the inclusion criteria. After PSM, there were 200 patients included. Multivariate analyses identified OPNI as an independent prognostic marker that was associated with primary site, tumor size, mitotic index, tumor rupture, necrosis, and modified NIH risk classification. Low OPNI (< 42.6; HR 0.409; *P* < 0.001) was associated with worse RFS.

**Conclusions:**

Preoperative OPNI is a novel and useful prognostic marker for GISTs both treated and not treated with TKIs. Higher NLR and PLR have negative effects on RFS.

## Introduction

Gastrointestinal stromal tumor (GIST) is the most common mesenchymal tissue neoplasm of the digestive system. It often occurs in the stomach and small intestine and is accidentally found in the abdomen and pelvis, omentum, colorectum, esophagus, pancreas, and elsewhere [1]. According to the literature, the incidence of GIST is approximately 0.001 ~ 0.0015% [2], which only accounts for a small proportion of gastrointestinal tumors. Elderly patients are more likely to have GIST. GISTs are now considered to originate in the interstitial cells of Cajal, and the most common cause is mutations in receptor tyrosine kinases, especially among adults with tumors expressing proto-oncogene c-kit and platelet-derived growth factor receptor A (PDGFRA) [3]. Treatment methods for GISTs are relatively limited because they are not sensitive to radiotherapy or chemotherapy, so surgical resection is the first choice and is the only potentially curative therapy. Tyrosine kinase inhibitors (TKIs) are used as routine clinical drugs for GIST patients of medium to high risk due to their strong effects [4]. Despite the availability of TKIs such as imatinib mesylate (IM), which greatly promotes disease-free survival (DFS), relapse of GIST is common, even when the tumors undergo R0 resection. Therefore, GIST is not easy to manage, and side effects are prevalent (fatigue, diarrhea, nausea, periorbital edema, muscle spasm, rash, etc.) and resistance to IM. Additionally, approximately 15% of GIST patients are innately resistant or intolerant to first-line imatinib treatment [5–7]. Therefore, accurate risk classification schemes are becoming increasingly useful for screening out patients who are most likely to benefit from systematic IM therapy. Currently, four widely accepted factors that can reflect the prognosis of GIST patients are tumor location, size, mitotic index and tumor rupture, as suggested by the National Institutes of Health (NIH) consensus criteria [8], the Armed Forces Institute of Pathology (AFIP) criteria [9], and the modified NIH consensus criteria [10]. Over time, more independent prognostic factors have been proposed, such as antigens identified by the monoclonal antibody Ki-67 index and surgical options [11, 12].

In addition, tumor-associated inflammatory cells, which dwell in the tumor microenvironment, promote the proliferation, invasion, and metastasis of tumor cells, thus enhancing the development and progression of tumors [13]. As many studies have shown, GISTs are also affected by immunoinflammatory factors such as the peripheral blood neutrophil-to-lymphocyte ratio (NLR) and platelet-to-lymphocyte ratio (PLR) [14], which are readily measurable, reproducible, and inexpensive systemic inflammatory markers. High NLR or PLR levels were reported to be associated with poor prognosis in various solid tumors. However, investigations on the prognostic value of NLR and PLR for GISTs are lacking, and the results remain controversial [15–17]. Onodera’s prognostic nutritional index (OPNI) was initially used to evaluate the immune-nutritional state of patients who are given gastrointestinal surgery [18]. Several studies have shown that OPNI is a crucial prognostic factor in some specific human cancers, such as gastric cancer [19], pancreatic cancer [20], colorectal cancer [21], and esophageal cancer [22]. Recently, an article about OPNI and GISTs illustrated that OPNI plays a crucial role in the prognostic prediction of GISTs that were not treated medicinally [23]. However, whether OPNI is a prognostic marker for GISTs treated with TKIs has not been clarified, and the predictability difference between GISTs treated with and without TKIs remains unknown. This study was the first to investigate these issues.

## Methods

### Patients

We retrospectively retrieved 563 cases of GIST ranging from the lowest to high risk according to the modified NIH risk classification at Nanjing Drum Tower Hospital from January 2010 to December 2018. Among the patients, 349 were not treated with TKIs, and the other 214 received TKI therapy. In this study, we selected patients classified as having intermediate and high risk and divided them into two groups: the TKI-using group and the TKI-non-using group. We intended to investigate whether OPNI can be a prognostic marker in these two groups. The inclusion criteria were as follows: (1) intermediate to high risk according to the modified NIH risk classification; (2) primary localized GIST with R0 resection; (3) no other synchronous primary tumors; (4) complete medical records; and (5) patient follow-up. Eventually, 280 GISTs were enrolled in this investigation. Among them, 102 patients received no imatinib, while 178 patients were treated with imatinib after the operation. This study was approved by the Ethics Committee of Nanjing Drum Tower Hospital. Written informed consent was acquired from all the patients in this program.

### Preoperative peripheral blood routine tests and OPNI evaluation

All the results of preoperative peripheral blood routine and blood biochemistry were obtained within 5 days before surgery. The NLR value was calculated as the neutrophil count (10^9^/L) divided by the lymphocyte count (10^9^/L). The platelet-to-lymphocyte ratio (PLR) was calculated in the same way. The OPNI was calculated as serum albumin (g/L) + 5 × total lymphocyte count (10^9^/L).

### Clinicopathological features

All GISTs were initially diagnosed as gastrointestinal mesenchymal tumors by pathological methods based on a combination of histopathological evaluation and immunohistochemistry for CD117 or Discovered On GIST 1 (DOG1). They were further confirmed by CD34, desmin, SMA, and S-100 expression. DNA mutation analysis of PDGFRA gene exons 12 and 18 or c-kit gene exons 9, 11, 13, and 17 was also performed partly to determine the application of TKIs. Clinical data and histopathological parameters were all collected from medical records. Clinical data included age, sex, initial complaint, primary tumor site, tumor size, surgery options, tumor rupture (preoperative or intraoperative), whether TKIs were used, and hospitalization time. Tumor size was accurately measured by pathologists after surgery. Histopathological factors included predominant cell type (spindle, epithelioid, or mixed), mitotic index (per 50 randomly selected high-power fields [HPFs]), tumor necrosis factor and Ki-67 index. Risk stratification of each case was determined by the modified NIH consensus criteria covering tumor size, mitotic index, tumor site, and rupture.

### Follow-up

After surgery, the patients were followed up through routine peripheral blood tests, abdominal ultrasonography, endoscopy, and computed tomography (CT) every 6 months in the first 5 years and then annually to evaluate tumor recurrence or distant metastasis. Follow-up information was obtained by outpatient or hospital records or direct communication with patients or their family. All of the clinical data and follow-up work was accomplished by professional staff in our department. Relapse-free survival (RFS) is more suitable to evaluate patient survival than overall survival (OS). RFS was calculated from the date of surgery to the date of GIST relapse, metastasis or last follow-up. The median follow-up time was estimated by the Kaplan–Meier method.

### Statistical analysis

All statistical analyses were calculated by using IBM SPSS Statistics, version 22.0 (IBM, New York, USA) and R 3.6.3. The ranked and unordered categorical variables, respectively, were assessed by the Mann–Whitney *U* test and the chi-square test. The correlation of continuous variables was calculated by the Pearson correlation coefficient, while the correlations between discrete variables were calculated by Spearman’s correlation coefficient. Cox’s regression model was used to perform multivariate survival analyses. The log-rank test and Kaplan–Meier method were utilized to calculate univariate survival. The PLR, NLR, and OPNI cut-off values were determined by R 3.6.3, which was performed based on the recurrence state at the 9-year follow-up. A *P* value < 0.05 indicated statistical significance, and confidence intervals (CIs) were calculated at the 95% level.

In this study, we applied 1:1 propensity score matching to match patients for sex, age, primary tumor site, tumor size, mitotic index, and risk stratification in order to reduce the effect of potential confounding factors and selection bias, such as patients’ baseline clinicopathologic factors or unequal patient distribution between the TKI-using and TKI-non-using groups. We applied the nearest neighbor matching method, and a caliper width of 0.5 standard deviations of the logit was set to match the two groups.

## Results

The median age of 280 patients was 60 years (range 26 to 83 years old), with 114 patients (40.7%) aged > 60 years. Among them, there were 143 men and 137 women. Primary manifestations of GISTs were as follows: abdominal discomfort or pain (*n* = 65), GI bleeding (*n* = 56), obstruction (*n* = 17), tumor perforation or rupture (*n* = 24), medical examination reported (*n* = 104), and other symptoms (*n* = 14). The primary tumor sites were mainly the stomach (*n* = 182), followed by the small intestine (*n* = 84) and colorectum or intraperitoneally with unknown origin (*n* = 14). The tumor size varied from 1.0 to 30.0 cm (median, 7.5 cm). Histologically, the spindle cell type was most common (*n* = 162), followed by the epithelioid cell type (*n* = 12) and mixed type (*n* = 6). The mitotic index, necrosis, and more detailed clinicopathological variables of our patients before and after PSM are summarized in Table 1.

**Table 1:** Clinicopathological features of 280 patients with primary GIST that classified as medium and high risk

**Characteristics**	**Before matching (*n* = 280)**			**After matching (*n* = 200)**		
	TKIs-used group(*n* = 178)	TKIs-unused group(*n* = 102)	*P* value	TKIs-used group(*n* = 100)	TKIs-unused group(*n* = 100)	*P* value
**Gender**			0.446			0.257
Male(%)	100(56.2)	43(42.2)		52(52.00)	43(43.00)	
Female(%)	78(43.8)	59(57.8)		48(48.00)	57(57.00)	
**Age (years)**			0.064			
≤ 60 years(%)	116(65.2)	50(49.0)		52(52.00)	50(50.00)	
> 60 years(%)	62(34.8)	52(51.0)		48(48.00)	50(50.00)	
**Clinical manifestation**			0.469			0.362
Abdominal discomfort or pain(%)	41(23.03)	24(23.53)		36(36.00)	39(39.0)	
Gastrointestinal bleeding(%)	36(20.22)	20(19.61)		17(17.00)	19(19.00)	
Obstruction(%)	11(6.18)	6(5.88)		2(2.00)	2(2.00)	
Perforation or rupture(%)	14(7.87)	10(9.80)		8(8.00)	9(9.00)	
Medical examination reported(%)	68(38.20)	36(35.29)		32(32.00)	27(27.00)	
Others(%)	8(4.49)	6(5.88)		5(5.00)	4(4.00)	
**Preoperative laboratory variables**						
Hemoglobin (g/L, $\overline{x} \pm {\text{s}}$)	113.3 ± 22.3	108.5 ± 26.4	0.206	116.3 ± 25.6	108.3 ± 26.5	
White blood cell (10^9^/L, $\overline{x} \pm {\text{s}}$)	6.4 ± 2.5	6.7 ± 4.2	0.662	6.4 ± 2.5	6.7 ± 4.2	
Neutrophil count (10^9^/L, $\overline{x} \pm {\text{s}}$)	4.4 ± 3.2	4.5 ± 4.0	0.809	4.2 ± 2.3	4.6 ± 4.0	
Lymphocyte count (10^9^/L, $\overline{x} \pm {\text{s}}$)	1.5 ± 0.6	1.6 ± 1.4	0.397	1.6 ± 0.6	1.6 ± 1.4	
Platelet count (10^9^/L, $\overline{x} \pm {\text{s}}$)	230.4 ± 87.7	237.8 ± 103.3	0.616	226.6 ± 78.4	237.4 ± 104.4	
Albumin (g/L,$\overline{x} \pm {\text{s}}$	38.9 ± 4.3	38.9 ± 4.8	0.992	39.0 ± 4.0	38.9 ± 4.9	
NLR ($\overline{x} \pm {\text{s}}$)	4.0 ± 5.5	3.9 ± 5.1	0.939	3.5 ± 3.8	3.9 ± 5.1	
PLR ($\overline{x} \pm {\text{s}}$)	182.7 ± 136.1	194.2 ± 143.3	0.339	170.3 ± 121.6	193.9 ± 143.9	
OPNI ($\overline{x} \pm {\text{s}}$)	46.4 ± 5.7	46.4 ± 8.6	0.015	47.2 ± 5.7	46.4 ± 8.6	
**Primary tumor site**			< 0.001			0.137
Stomach(%)	114(64.04)	68(66.67)		75(75.00)	66(66.00)	
Small intestine(%)	54(30.34)	30(29.41)		20(20.00)	30(30.00)	
Colorectum(%)	3(1.69)	2(1.96)		2(2.00)	2(2.00)	
Intraperitoneally with unknown origin(%)	7(3.93)	2(1.96)		3(3.00)	2(2.00)	
**Tumor size (cm,**$\overline{\user2{x}}{\mathbf{ \pm s}}$**)**	7.75 ± 3.64	7.17 ± 4.45	0.051	6.67 ± 2.75	7.16 ± 4.49	0.469
≤ 5.0(%)	32(17.98)	27(26.47)		23(23.00)	26(26.00)	
5.1–10.0(%)	112(62.92)	62(60.78)		72(72.00)	61(61.00)	
> 10.0(%)	34(19.10)	13(12.75)		5(5.00)	13(13.00)	
**Predominant cell type**			0.685			0.795
Spindle(%)	170(19.50)	92(90.30)		95(95.00)	90(90.00)	
Epithelioid(%)	5(2.81)	7(6.86)		4(4.00)	7(7.00)	
Mixed (%)	3(1.69)	3(2.94)		1(1.00)	3(3.00)	
**Mitotic index (per 50 HPFs)**			0.875			0.250
≤ 5(%)	85(47.75)	47(46.08)		53(53.00)	45(45.00)	
6–10(%)	35(19.66)	26(25.49)		21(21.00)	26(26.00)	
> 10(%)	58(32.58)	29(28.43)		26(26.00)	29(29.00)	
**Necrosis**			0.014			0.002
Yes (%)	62(34.83)	32(31.37)		33(33.00)	37(37.00)	
No (%)	116(65.17)	70(68.63)		67(67.00)	63(63.00)	
**Tumor rupture**			< 0.001			< 0.001
Yes (%)	23(12.92)	14(13.73)		6(6.00)	7(7.00)	
No (%)	155(87.08)	88(86.27)		94(94.00)	93(93.00)	
**Risk classification**			0.096			0.024
Intermediate(%) risk	59(33.15)	44(43.14)		58(58.00)	42(42.00)	
High risk(%)	119(66.85)	58(56.86)		42(42.00)	58(58.00)	
**CD117**			0.279			0.031
(–) (%)	3(1.69)	5(4.90)		2(2.00)	5(5.00)	
( +)(%)	37(20.79)	27(26.47)		23(23.00)	25(25.00)	
(+ +)(%)	35(19.66)	10(9.80)		17(17.00)	11(11.00)	
(+ + +)(%)	103(57.87)	60(58.82)		58(58.00)	59(59.00)	
**CD34**			0.257			0.531
(–) (%)	20(11.24)	12(11.76)		10(10.00)	12(12.00)	
( +)(%)	33(18.54)	36(35.29)		15(15.00)	33(33.00)	
(+ +)(%)	22(12.36)	8(7.84)		12(12.00)	8(8.00)	
(+ + +)(%)	103(57.87)	46(45.10)		63(63.00)	46(46.00)	
**Ki-67 index (%,**$\overline{\user2{x}}{\mathbf{ \pm s}}$**)**	7.87 ± 7.66	7.31 ± 7.59	0.019	7.01 ± 6.62	7.35 ± 7.69	0.766
≤ 5(%)	110(61.80)	64(62.75)		67(67.00)	63(63.00)	
6–10(%)	35(19.66)	25(24.51)		16(16.00)	24(24.00)	
> 10(%)	33(18.54)	13(12.75)		17(17.00)	13(13.00)	
**Follow-up time** (months, $\overline{x} \pm {\text{s}}$)	44.47 ± 25.07	55.01 ± 29.39	0.003	48.57 ± 24.61	53.98 ± 28.75	< 0.001
**Follow-up status**			< 0.001			< 0.001
No relapse (%)	146(82.02)	72(70.59)		85(85.00)	70(70.00)	
Relapse (%)	32(17.98)	30(29.41)		15(15.00)	30(30.00)	

**Fig. 1:**
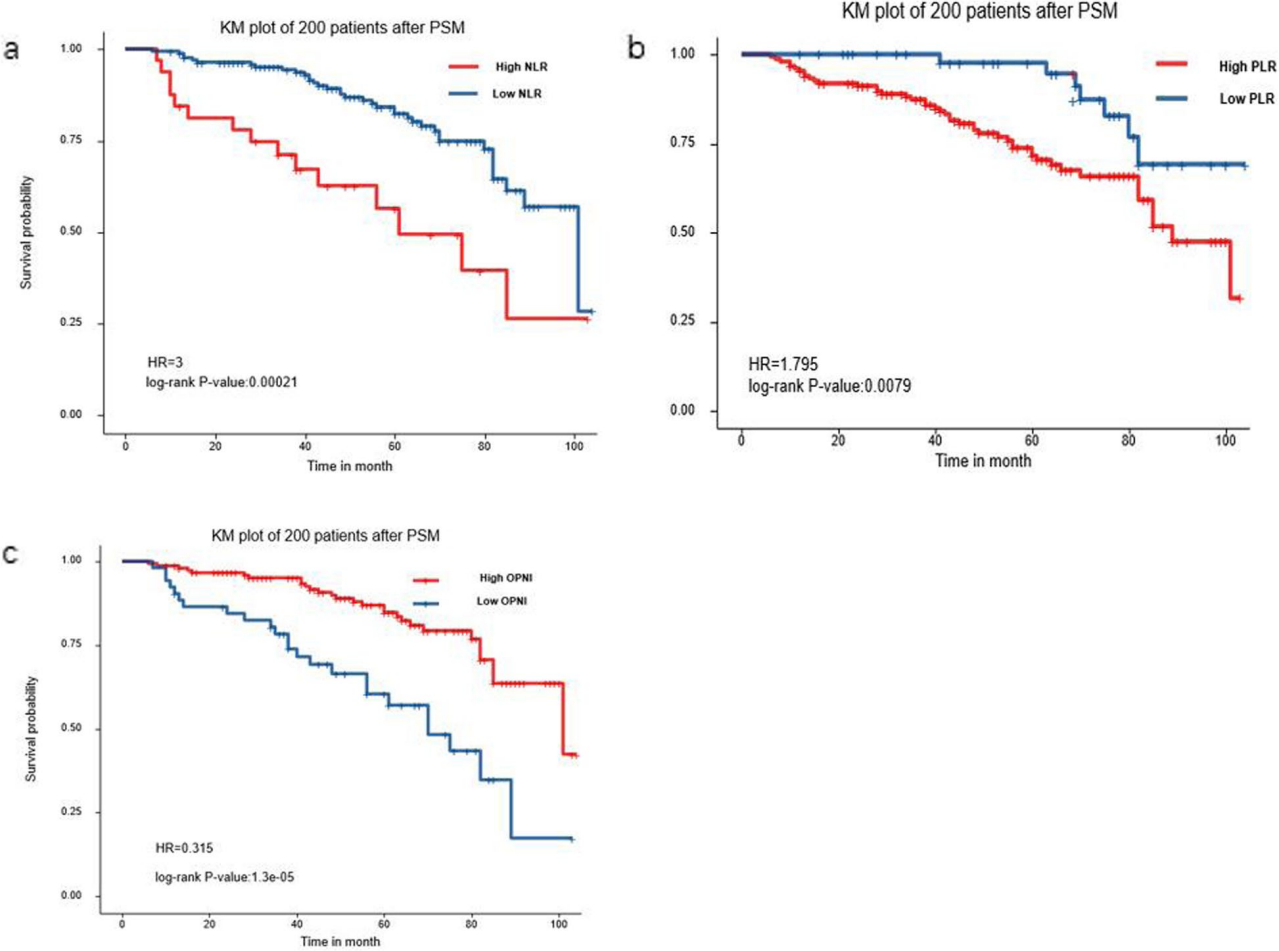
ROC analysis of NLR (**a**), PLR (**b**) and OPNI (**c**) in TKIs-unused patients. The PLR, NLR, and OPNI cut-off value was determined by R 3.6.3 which was performed based on the recurrence state at 9-year follow-up. And the cut-off point of OPNI is 42.1 (*P* < 0.001), NLR is 5.1(*P* < 0.001) and PLR is 98.6 (*P* = 0.008)

According to a recent study, OPNI is a prognostic marker for GIST [23]. We used the continuous variables NLR, PLR, and OPNI of 200 patients after PSM and their RFS and outcome as the state variables. The Hosmer–Lemeshow test and the value of the c-statistic (0.71) showed fairly excellent calibration (*p* = 0.08) and discrimination, respectively, between the 2 groups. The ASD values after matching ranged from 0 to 8%. The cut-off point of OPNI was 42.6 (*P* < 0.001), of NLR was 5.1 (*P* < 0.001), and of PLR was 98.6 (*P* = 0.008). Exp (coef), univariate *P* value and hazard ratio of NLR, PLR, and OPNI are summarized in Table 2 and Fig. 1.

**Table 2:** Analysis for NLR, PLR, and OPNI

	**NLR**	**PLR**	**OPNI**
**Exp(coef)**	1.038	1.002	0.933
**Univariate *P* value**	0.129	0.084	0.002
**Best cut-off point**	5.1	98.6	42.6
**Hazard ratio**	3.000	1.795	0.315
**Log rank *P***	< 0.001	0.008	< 0.001

**Fig. 2:**
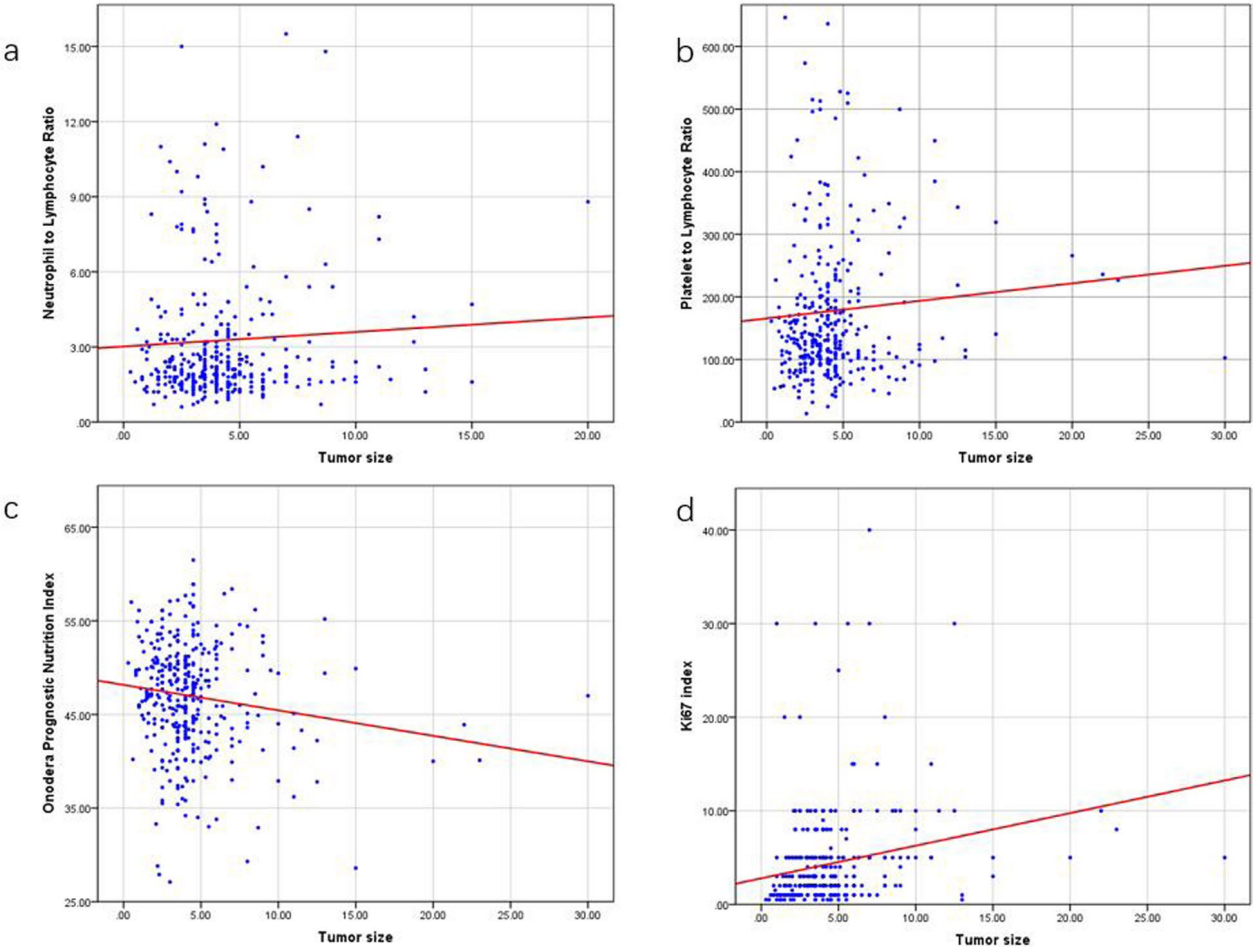
Correlation between tumor size and NLR (**a**), PLR (**b**), OPNI (**c**), and Ki-67 index (**d**). The correlation of continuous variables was calculated by Pearson correlation coefficient, while discrete variables by Spearman’s correlation coefficient. Higher OPNI was associated with primary tumor site of stomach (*P* < 0.01), smaller tumor size (*P* < 0.017), lower mitotic index (*P* < 0.001), lower modified NIH risk classification (*P* < 0.001), less gastrointestinal bleeding rate (*P* < 0.01) and tumor rupture (*P* < 0.01), and much lower tumor relapse rate (*P* < 0.01)

The Spearman correlation analysis showed that higher OPNI was associated with a primary tumor site of the stomach (*P* < 0.01), smaller tumor size (*P* < 0.017), lower mitotic index (*P* < 0.001), lower modified NIH risk classification (*P* < 0.001), less gastrointestinal bleeding rate (*P* < 0.01) and tumor rupture (*P* < 0.01), and much lower tumor relapse rate (*P* < 0.01). A strong correlation was observed between NLR and tumor site (*P* = 0.01), GI bleeding (*P* < 0.01), tumor rupture (*P* = 0.03), and relapse (*P* = 0.02). PLR was also connected with tumor site (P < 0.01), GI bleeding (*P* = 0.04) and relapse (*P* = 0.03) (Tables 3 and 4, Fig. 2).

**Table 3:** Correlation analysis of tumor size and mitotic index with NLR, PLR, OPNI, and Ki-67 index

	**Tumor size**		**Mitotic index**	
	**Spearman *r***	***P* value**	**Spearman r**	***P* value**
**NLR**	0.06	< 0.01	0.11	0.07
**PLR**	0.28	< 0.01	0.15	0.01
**OPNI**	− 0.27	< 0.01	− 0.14	0.02
**Ki-67 index**	0.35	0.02	0.44	< 0.01

**Table 4:** Correlation analysis of OPNI, NLR, and PLR with tumor site, gastrointestinal bleeding (GI bleeding), tumor rupture, relapse, CD34, and CD117

**Factors**	**OPNI**		**NLR**		**PLR**	
	**Spearman *r***	***P* value**	**Spearman *r***	***P* value**	**Spearman *r***	***P* value**
**Site**	− 0.26	< 0.01	0.18	0.01	0.19	< 0.01
**GI bleeding**	− 0.26	< 0.01	0.20	< 0.01	0.15	0.04
**Tumor rupture**	− 0.13	0.06	0.15	0.03	0.12	0.09
**Relapse**	− 0.23	< 0.01	0.16	0.02	0.15	0.03
**CD34**	− 0.07	0.26	0.02	0.74	0.07	0.27
**CD117**	− 0.06	0.36	− 0.01	0.81	0.05	0.38

Patients were followed for a median of 48 months (range 8 months–103 months). This was calculated by the Kaplan–Meier method, which concluded that our estimated median follow-up time was 47.98 months (*P* < 0.001). Sixty-two patients experienced tumor relapse during the follow-up period. Thirty of them had not been treated by IM, and 32 of them had received IM therapy with a duration ranging from 1 month to 5 years. Until December 2018, 13 patients without medication had died, for a mortality rate of 12.7% (13/102), while the mortality rate in the drug treatment group was 3.3% (6/178). Metastasis to the lymph nodes was not spotted.

Our univariate survival analysis showed that tumor size (log-rank *P* = 0.002), mitotic index (log-rank *P* < 0.001), modified NIH risk stratification (log-rank *P* < 0.001), NLR (log-rank *P* = 0.002), PLR (log-rank *P* = 0.004), primary tumor site (log-rank *P* = 0.007), age (log-rank *P* = 0.023), and OPNI (log-rank *P* = 0.045) were all significant prognostic parameters for RFS. The results of univariate survival analysis are shown in Table 5. Some sorted factors were analyzed in the Cox proportional hazards model by the enter strategy. The results of the Cox regression analysis are listed in Table 5. High mitotic index (*P* = 0.002), age greater than 60 (*P* = 0.008), larger tumor size (*P* = 0.003), high NLR (*P* = 0.031), and low OPNI (*P* = 0.009) were statistically significant, independent negative prognostic indicators for RFS.

**Table 5:** Univariate and multivariate analysis of the prognostic factors for recurrence-free survival of patients after PS matching

**Characteristics**	**Univariate analysis**		**Multivariate analysis**	
	HR(95% CI)	*P* value	HR(95% CI)	*P* value
**Age/year**		**0.023**		**0.008**
≤ 60	1		1	
> 60	1.910(1.095–3.333)		2.175(1.226–3.859)	
**Gender**		0.064		
Male	1			
Female	0.533(0.331–1.120)			
**GI bleeding**		0.337		
Yes	1			
No	1.307(0.756–2.261)			
**Primary site**		**0.007**		0.864
Gastric	1		1	
Non-gastric	2.116(1.228–3.645)		1.066(0.560–2.029)	
**Tumor size**		**0.002**		**0.003**
≤ 5.0 cm	1		1	
> 5.0 cm	1.962(1.254–2.848)		1.758(1.067–3.759)	
**Predominant cell type**		0.419		
Spindle	1			
Epithelioid	0.765(0.263–3.376)			
Mixed	0.735(0.363–3.289)			
**Mitotic index**		** < 0.001**		**0.002**
≤ 5 per 50 HPFs	1		1	
6–10 per 50 HPFs	1.524(1.186–2.654)		1.387(1.084–2.368)	
> 10 per 50 HPFs	2.098(1.522–2.890)		1.811(1.247–2.629)	
**Tumor rupture**		0.097		
No	1			
Yes	1.801(0.711–4.563)			
**NIH risk classification**		** < 0.001**		**0.024**
Intermediate risk	1		1	
High risk	4.943(2.479–9.854)		2.640(1.140–6.115)	
**Ki-67 index**		0.181		
≤ 5	1			
6–10	1.128(0.581–3.835)			
> 10	1.045(0.854–4.185)			
**NLR**		**0.002**		**0.031**
< 5.1	1		1	
≥ **5.1**	1.552(1.180–2.041)		1.878 (1.060–3.327)	
**PLR**		**0.004**		0.407
< 98.6	1		1	
≥ **98.6**	1.453(1.126–1.875)		1.262(0.727–2.191)	
**OPNI**		**0.045**		**0.009**
< 42.6	1		1	
≥ **42.6**	0.409(0.170–0.980)		0.592 (0.400–0.875)	

**Fig. 3:**
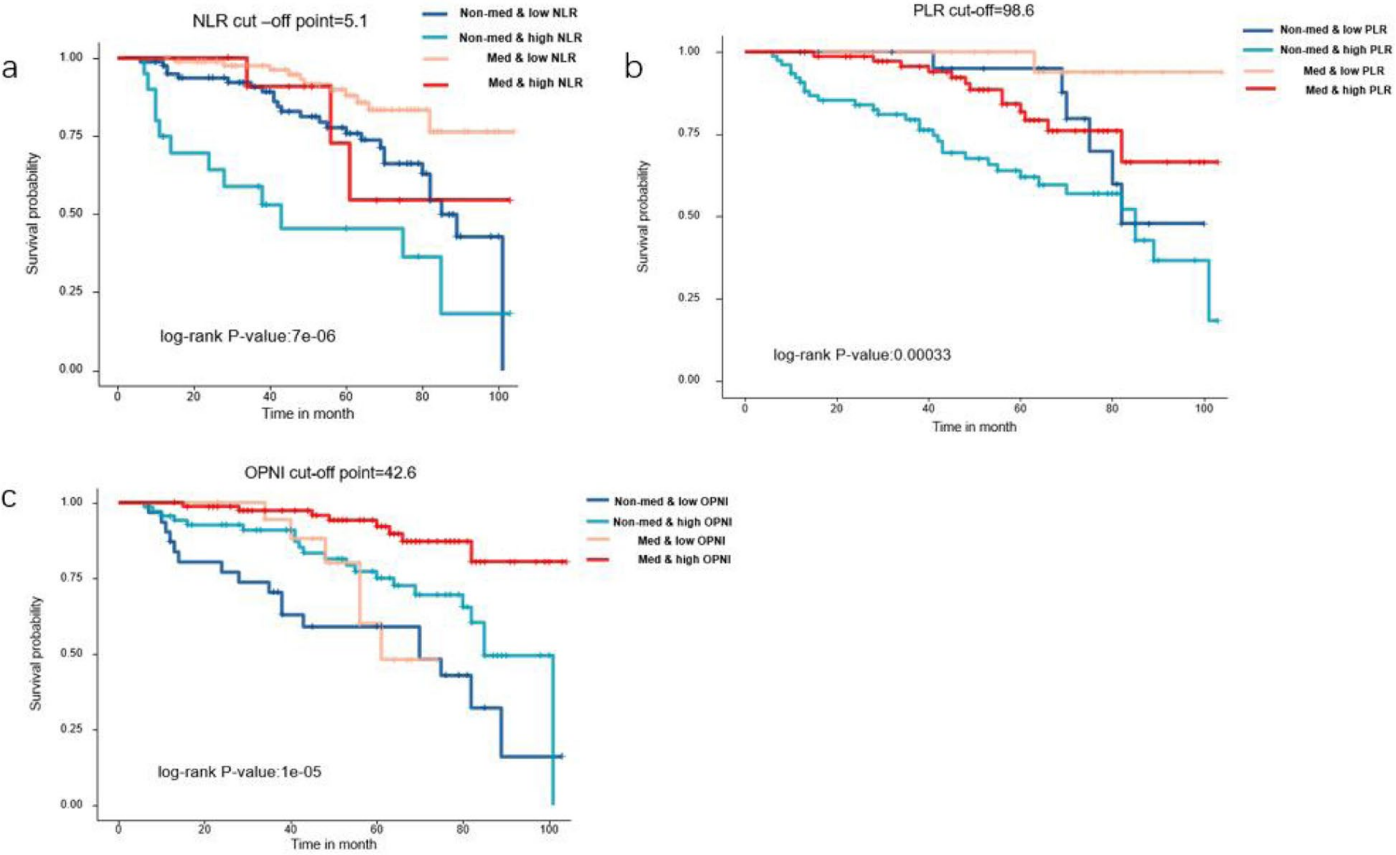
Recurrence-free survival analysis of 200 patients after PSM. Kaplan–Meier curve analysis demonstrated a worse relapse-free survival for patients presenting with **a** higher NLR, **b** higher PLR, and **c** lower OPNI. Patients treated with TKIs had better prognosis in our study and low NLR, low PLR, and high OPNI also indicated better prognosis

We divided patients with or without medical treatment into several groups according to their relatively low or high OPNI, NLR, and PLR. We observed that patients who used TKIs had better relapse-free survival. In addition, patients with higher OPNI showed longer RFS than those with lower OPNI (*P* < 0.0001). Furthermore, higher NLR (*P* < 0.0001) and PLR (*P* = 0.0003) were factors leading to poor prognosis (Fig. 3).

## Discussion

According to recent investigations by Sun JY’s team, OPNI was an independent predictive factor of RFS in GIST patients with no TKI treatment [23]. We initially based our survival cut-off analysis on the 200 GIST patients with median or high risk after PSM to find the best cut-off points of NLR, PLR, and OPNI. Then, we examined the univariate and multivariate survival analyses of our patients after PSM. Our aim was to investigate the prognostic value of OPNI in intermediate- and high-risk gastrointestinal stromal tumors treated with or without TKIs. In the end, the analysis showed that OPNI was an independent prognostic marker for both groups.

A more precise risk classification criterion that can be applied to determine the postoperative prognosis of patients with GIST is urgently required. The items should be simply and economically detected and calculated from clinicopathological data. Currently, the most widely used criteria to estimate the risk of relapse after surgery in GIST are the AFIP criteria and modified NIH consensus criteria. Studies have found that their prognostic accuracy is similar [24]. Moreover, the Memorial Sloan-Kettering Cancer Center sarcoma team developed a nomogram that could estimate the probability of RFS at 2 and 5 years after surgery for primary GIST and was more precise than the NIH criteria to a certain extent [25]. Joensuu H further demonstrated that KIT and PDGFRA mutations may bring widely varying risks for recurrence, and those with KIT exon 11 duplication mutations or deletion of one codon have favorable RFS with surgery alone [26].

OPNI is a nutrition index that was first proposed by Onodera and his colleagues. A previous study showed that patients with high OPNI shared a significantly better prognosis than those who had a lower value of OPNI [22]. Similar results regarding Crohn’s disease and stage III colorectal cancer have also been reported [1, 27, 28]. In our study, the border value of the OPNI was determined to be 42.6 for the TKI-unused group and TKI-unused group according to the survival cut-off analysis in R 3.6.3. A detailed analysis demonstrated that higher OPNI was associated with a primary tumor site of the stomach, smaller tumor size, lower mitotic index, lower modified NIH risk classification, better gastrointestinal bleeding rate and tumor rupture, and much lower tumor relapse rate. In univariate and multivariate survival analyses, OPNI was also an independent prognostic indicator. Lower OPNI may result from low hypoproteinemia and/or lymphopenia, which can be explained by several potential phenomena: (1) nutritional supplementation with branched-chain amino acids can improve hypoproteinemia and reduce tumor recurrence in patients; and (2) lymphocytes play an important role in the host immune response, eliminating tumor formation and progression. Postoperative follow-up examination for OPNI is also recommended, though this study lacked these data.

In our univariate and multivariate analysis of the prognostic factors for recurrence-free survival of patients after PS matching, we found that high mitotic index, age more than 60 years, larger tumor size, high NLR, and low OPNI were statistically significant independent negative prognostic indicators for RFS. Moreover, when we split the patients into groups according to the best cut-off values of NLR, PLR, and OPNI, we found that relatively lower NLR, lower PLR, and higher OPNI were significantly linked to better RFS.

There were limitations of this study. First, it was a single-center retrospective study, so a multicenter study is urgently required to enlarge the sample to minimize the deficiencies of the analysis. Second, the best cut-off value in this study was determined by survival cut-off analysis. However, it is still unclear what cut-off value is the best optimal cut-off value for the clinical diagnosis of GIST due to the limited number of patients. Third, mutations were not well considered because only some patients underwent gene tests. Exploring the exact best cut-off value and studying its intrinsic molecular mechanism will be our future research directions.

In conclusion, we found connections among immunoinflammatory factors (NLR and PLR), nutritional factors (OPNI), clinicopathological characteristics and the RFS of intermediate- and high-risk GISTs treated with or without TKIs. OPNI is an independent indicator for RFS in GISTs treated with or without TKIs. Furthermore, OPNI might also be a valuable factor for predicting tumor biological behavior from peripheral blood.

## Data Availability

Access to the data and the calculation method can be obtained from the authors by email (fengwang36@163.com).

## Abbreviations

NLR: Neutrophil-to-lymphocyte ratio
PLR: Platelet-to-lymphocyte ratio
OPNI: Onodera’s prognostic nutritional index
GIST: Gastrointestinal stromal tumors
PSM: Propensity score matching
RFS: Recurrence-free survival
TKIs: Tyrosine kinase inhibitors
IM: Imatinib mesylate
NCCN: National Comprehensive Cancer Network
NIH: National Institutes of Health
AFIP: Armed Forces Institute of Pathology
DFS: Disease-free survival
OS: Overall survival
PDGFRA: Platelet-derived growth factor receptor A
HPFs: High power fields
CT: Computed tomography
ROC: Receiver operating characteristic
CI: Confidence intervals
